# Pediatric Tuberculosis in Italian Children: Epidemiological and Clinical Data from the Italian Register of Pediatric Tuberculosis

**DOI:** 10.3390/ijms17060960

**Published:** 2016-06-17

**Authors:** Luisa Galli, Laura Lancella, Chiara Tersigni, Elisabetta Venturini, Elena Chiappini, Barbara Maria Bergamini, Margherita Codifava, Cristina Venturelli, Giulia Tosetti, Caterina Marabotto, Laura Cursi, Elena Boccuzzi, Silvia Garazzino, Pier Angelo Tovo, Michele Pinon, Daniele Le Serre, Laura Castiglioni, Andrea Lo Vecchio, Alfredo Guarino, Eugenia Bruzzese, Giuseppe Losurdo, Elio Castagnola, Grazia Bossi, Gian Luigi Marseglia, Susanna Esposito, Samantha Bosis, Rita Grandolfo, Valentina Fiorito, Piero Valentini, Danilo Buonsenso, Raffaele Domenici, Marco Montesanti, Filippo Maria Salvini, Enrica Riva, Icilio Dodi, Francesca Maschio, Luisa Abbagnato, Elisa Fiumana, Chiara Fornabaio, Patrizia Ballista, Vincenzo Portelli, Gabriella Bottone, Nicola Palladino, Mariella Valenzise, Barbara Vecchi, Maria Di Gangi, Carla Lupi, Alberto Villani, Maurizio de Martino

**Affiliations:** 1 Department of Health Sciences, University of Florence, Pediatric Infectious Diseases Division, Anna Meyer Children’s University Hospital, Florence 50139, Italy; chia88.te@gmail.com (C.T.); elisabetta-venturini@virgilio.it (E.V.); elena.chiappini@unifi.it (E.C.); 2 Unit of General Pediatrics and Pediatric Infectious Diseases, Istituto di Ricovero e Cura a Carattere Scientifico (IRCCS) Bambino Gesù Hospital, Rome 00165, Italy; laura.lancella@opbg.net (L.L.); caterina.marabotto@gmail.com (C.M.); laura.cursi@opbg.net (L.C.); elena.boccuzzi@opbg.net (E.B.); alberto.villani@opbg.net (A.V.); 3 Department of Pediatrics, University of Modena and Reggio Emilia, Modena 41121, Italy; bergamini.barbaramaria@unimo.it (B.M.B.); mar.cody@libero.it (M.C.); criventurelli@alice.it (C.V.); giuliatosetti@hotmail.com (G.T.); 4 Pediatric Infectious Diseases Unit, Regina Margherita Hospital, University of Turin, Turin 10126, Italy; silvia.garazzino@unito.it (S.G.); pierangelo.tovo@unito.it (P.A.T.); michele.pinon@unito.it (M.P.); daniele.leserre@gmail.com (D.L.S.); 5 Pediatric Unit, Ramazzini Hospital, Carpi (Modena) 41124, Italy; l.castiglioni@ausl.mo.it; 6 Section of Pediatrics, Department of Translational Medical Science, University of Naples Federico II, Naples 80131, Italy; andrea.lovecchio@unina.it (A.L.V.); alfguari@unina.it (A.G.); eugbruzz@unina.it (E.B.); 7 Infectious Diseases Unit, IRCCS Giannina Gaslini, Genoa 16148, Italy; giuseppelosurdo@ospedale-gaslini.ge.it (G.L.); eliocastagnola@ospedale-gaslini.ge.it (E.C.); 8 Pediatric Clinic, University of Pavia, IRCCS Policlinico “S. Matteo” Foundation, Pavia 27100, Italy; g.bossi@smatteo.pv.it (G.B.); gl.marseglia@smatteo.pv.it (G.L.M.); 9 Pediatric Highly Intensive Care Unit, Department of Pathophysiology and Transplantation, Università degli Studi di Milano, Fondazione IRCCS Ca’ Granda Ospedale Maggiore Policlinico, Milan 20122, Italy; susanna.esposito@unimi.it (S.E.); samantha.bosis@unimi.it (S.B.); 10 Infectious Diseases Unit, St. Giovanni XXIII Pediatric Hospital, Bari 70120, Italy; ritagrando@yahoo.it; 11 Pediatric Unit, S. Maria del Carmine Hospital, Rovereto (Trento) 38068, Italy; valentina.fiorito@apss.tn.it; 12 Institute of Pediatrics, Università Cattolica del Sacro Cuore, Rome 00168, Italy; pvalentini@rm.unicatt.it (P.V.); danilobuonsenso@gmail.com (D.B.); 13 Pediatric Unit, “Campo di Marte” Hospital, Lucca 55100, Italy; r.domenici@usl2.toscana.it (R.D.); m.montesanti@usl2.toscana.it (M.M.); 14 Pediatric Clinic, San Paolo Hospital, University of Milan, Milan 20142, Italy; filippo.salvini@ao-sanpaolo.it (F.M.S.); enrica.riva@unimi.it (E.R.); 15 Department of Pediatrics, Parma University Hospital, Parma 43126, Italy; idodi@ao.pr.it; 16 Pediatric Unit, Ca’ Foncello Civil Hospital, Treviso 31100, Italy; fmaschio@ulss.tv.it; 17 Pediatric Unit, St. Anna Hospital, Como 22100, Italy; luisa.abbagnato@libero.it; 18 Pediatric Unit, Arcispedale Sant’Anna, University of Ferrara, Ferrara 44100, Italy; efiumana@virgilio.it; 19 Division of Infectious Diseases, Istituti Ospitalieri di Cremona, Cremona 26100, Italy; c.fornabaio@ospedale.cremona.it; 20 Pediatric Unit, Sesto San Giovanni Hospital, Milan 20099, Italy; patrizia.ballista@icp.mi.it; 21 Infectious Diseases Unit, S. Antonio Abate Hospital, Trapani 91016, Italy; vincenzoportelli@asptrapani.it; 22 Department of Internal Medicine and Public Health, University of L’Aquila, L’Aquila 67100, Italy; gbottone@asl1abruzzo.it; 23 Infectious Disease Section, Department of Experimental Medicine and Biochemical Sciences, University of Perugia, Perugia 06100, Italy; nicola.palladino@asl1.umbria.it; 24 Department of Pediatrics, Gynecology, Microbiological and Biomedical Sciences, University of Messina, Messina 98126, Italy; marielvale@hotmail.com; 25 Pediatric Infectious Disease Unit, Narni Hospital, Narni (Terni) 05035, Italy; barbara.vecchi@alice.it; 26 Infectious Disease Section, Palermo-Civico Hospital, Azienda di Rilievo Nazionale ad Alta Specializzazione (ARNAS), Palermo 90127, Italy; digangim@libero.it; 27 Pediatric clinic, Santa Maria della Misericordia Hospital, Perugia 06156, Italy; carlalupi@hotmail.it; 28 Department of Health Sciences, University of Florence, Anna Meyer Children’s University Hospital, Florence 50139, Italy; maurizio.demartino@unifi.it

**Keywords:** tuberculosis, children, Italian, register

## Abstract

Tuberculosis (TB) is one of the leading causes of death worldwide. Over the last decades, TB has also emerged in the pediatric population. Epidemiologic data of childhood TB are still limited and there is an urgent need of more data on very large cohorts. A multicenter study was conducted in 27 pediatric hospitals, pediatric wards, and public health centers in Italy using a standardized form, covering the period of time between 1 January 2010 and 31 December 2012. Children with active TB, latent TB, and those recently exposed to TB or recently adopted/immigrated from a high TB incidence country were enrolled. Overall, 4234 children were included; 554 (13.1%) children had active TB, 594 (14.0%) latent TB and 3086 (72.9%) were uninfected. Among children with active TB, 481 (86.8%) patients had pulmonary TB. The treatment of active TB cases was known for 96.4% (*n* = 534) of the cases. Overall, 210 (39.3%) out of these 534 children were treated with three and 216 (40.4%) with four first-line drugs. Second-line drugs where used in 87 (16.3%) children with active TB. Drug-resistant strains of *Mycobacterium tuberculosis* were reported in 39 (7%) children. Improving the surveillance of childhood TB is important for public health care workers and pediatricians. A non-negligible proportion of children had drug-resistant TB and was treated with second-line drugs, most of which are off-label in the pediatric age. Future efforts should concentrate on improving active surveillance, diagnostic tools, and the availability of antitubercular pediatric formulations, also in low-endemic countries.

## 1. Introduction

Tuberculosis (TB) is one of the leading causes of death worldwide [1]. Global international migration flows, Human Immunodeficiency Virus (HIV)-related immunodeficiency, immunosuppressive therapies and the spread of multidrug-resistant (MDR) strains have been recognized as the most important factors leading to an increased TB incidence [2]. Epidemiologic data on childhood TB are still limited. In 2013, the WHO estimated nine million (range 8.6–9.4 million) new TB cases/year, equivalent to 126 cases per 100,000 populations, with about 6% of those cases occurring in children [1]. The estimation methods presently have some limitations including misdiagnosis due to difficulties in childhood TB diagnosis, under-reporting and unavailability of age-disaggregated data. In 2013 the total number of new and relapsed cases among children was 275,000 in countries that reported age-disaggregated notification data for 2013 [1]. In Europe, 2625 TB cases in children below age 15 were reported in 2013, accounting for 4% of all notified TB cases [3]. Romania, Spain and the United Kingdom accounted for more than half of the cases. In 2013, the total number of active TB-notified cases in Italian children (0–14 years) was 124, corresponding to 4.7% of the European cases in the same year [3].

Childhood TB is considered as a sentinel of disease spreading throughout the community [2]. Furthermore, children are particularly vulnerable to severe disease and death following a TB infection, and those with a latent infection could become a reservoir of disease reactivation in adulthood [2]. As a result, pediatric TB deserves prompt identification. Although local databases have been implemented in single institutions [4,5,6,7], this is the first Italian nationwide Register on Pediatric Tuberculosis.

## 2. Results

Children with active TB, latent TB, and those recently exposed to TB or recently adopted/immigrated from a high TB incidence country. Overall, 4234 children were included from 27 centers. According to the infection status, 554 (13.1%) children had active TB, 594 (14.0%) latent TB and 3086 (72.9%) were uninfected. The anagraphic data and the main characteristics of the children enrolled are reported in Table 1.

Overall, 21.6% of the patients enrolled were Italian. Children of foreign origins were more frequently from Asia, Eastern Europe and South America, and the same distribution was found in all groups.

Overall, a BCG (Bacillus Calmette–Guerin) vaccination status was reported only in 74% of children.

### 2.1. Uninfected Children

Considering the 3086 uninfected children, 2782 (90.1%) had a response to TST ≤ 5 mm, whereas 304 had a TST > 5 mm (9.9%). Among these, 294/304 (96.7%) had a negative IGRA test; the IGRA result was unknown in seven (1.8%) and indeterminate in three (0.8%) children. One hundred sixty-one children were screened for TB because of symptoms/signs. The most common clinical features were lymphadenopathy in 45 (27.9%) cases and fever in 15 (9.3%) cases. The median age of these children was 68 (IQR: 38.5–123) months.

### 2.2. Children with Tuberculosis (TB)

The main characteristics of children with TB are reported in Table 1. As shown, children with active TB were significantly younger compared to those with latent TB (*p* < 0.001). Moreover, active TB patients were often investigated because they were symptomatic or recently exposed to a TB case (521/553, 94.2%), whereas children with latent TB were mainly investigated for screening purposes (359/594, 60.4%). A significantly higher proportion of Italian children were affected by active compared to latent TB (27.4% *vs.* 11.8%; 95% CI: 10.9–20.2; *p* < 0.0001).

Among 541 children with active TB for whom both birthplace and country of origin were known (of the child if born abroad or of the parents), 141 (26.8%) were born in Italy from Italian parents, 240 (44.4%) were born in Italy from foreign parents and 156 (28.8%) were born abroad from foreign parents.

A significantly higher proportion of children with latent TB were vaccinated with BCG compared to active TB cases (49.2% *vs.* 11.3%; 95% CI: 32.8–42.7; *p* < 0.001). No statistically significant difference in BCG vaccination was found between pulmonary and extra-pulmonary TB cases (*p* = 0.118).

Among 554 children with active TB, 481 (86.8%) patients had pulmonary TB (Table 2).

In 49 children, the disease also involved other sites besides the lungs. Extra-pulmonary TB was diagnosed in 122 children; of those, 41.8% children had lymphonodal TB, 18% central nervous system (CNS) TB (meningitis in 20 cases and two intracranial tuberculoma), 19.7% bone TB, and 28.7% had other sites involved (*i.e.*, abdominal, cardiac, ocular or disseminated TB). The distribution of TB localization by to age groups is reported in Table 3.

CNS TB resulted as being significantly more frequent in children under age four (*p* < 0.009). In contrast, lymphonodal TB was more frequent in older children (*p* < 0.003). Two children died from cardio-pulmonary complications.

### 2.3. Diagnosis

In the active TB group, 396 children were symptomatic at presentation (396/554, 71.5%) (Table 2). Within the pulmonary TB group, the more common signs/symptoms were cough (52/181, 28.7%), fever (65/181, 35.9%) and weight loss (18/181, 9.9%). Hemoptysis was reported in five cases (5/181, 2.7%). The more frequent clinical manifestations regarding CNS TB were fever (16/22, 72.7%) and signs/symptoms of CNS involvement (seizures, strabismus, diplopia, and headache) (11/22, 50%).

Overall, TST was performed in all the children enrolled in the study. Within the active TB group, TST was negative in 14.8% children, whereas IGRA was negative in 8.3% of the cases and indeterminate in eight patients (Table 4).

Sensitivity of TST and QFT-G-IT in the diagnosis of active TB was 85.2% (95% CI: 81.9–88) and 91.5% (95% CI: 88.4–93.9), respectively. Specificity was 97.3% (95% CI: 93.4–99.2) and 98% (95% CI: 94.2–99.5), respectively.

Imaging was performed using chest X-rays in 477 active TB cases (477/554; 86.1%) and chest computer tomography (CT) in 309 cases (309/554, 55.8%), of whom about half of the cases (148/309, 47.9%) with contrast. Other diagnostic imaging was performed using lymphonodal or abdominal ultrasound in 16 children (16/554, 2.9%) and magnetic resonance imaging in 21 cases (21/554, 3.8%).

Respiratory samples were gathered from all children with pulmonary TB. Overall, respiratory samples by gastric aspirates, sputum or bronchoalveolar lavage (BAL) were collected in 451/554 (81.4%) active TB cases. Gastric aspirates were collected in 388 active TB cases (388/554, 70%), with a median age of 40 months (IQR: 16.2–105.7). In 95.6% (*n* = 371) of the cases, the culture result was known, being positive in 152 children (152/371, 40.9%). The Ziehl–Neelsen (ZN) staining was known for 377 patients (97.1%), being positive only in 56 children (56/377, 14.8%). The molecular investigation by polymerase chain reaction (PCR) was positive in one-third of the cases (94/309, 30.4%). Considering the culture as the gold standard for the diagnosis, the sensitivity and specificity of PCR in our population were 59% (95% CI: 49.6–68.4; 67/113) and 90% (95% CI: 85–94.3; 160/177), respectively. The sensitivity and specificity of ZN were 31% (95% CI: 23.9–39.4; 46/147) and 97% (95% CI: 93.9–98.9; 206/212), respectively.

Sputum was performed in 55 children (55/554, 9.9%), aged 149 (IQR: 97.5–175) months.

Results for culture, ZN and PCR were available in 38 children (38/55, 69%). More than one-third of the cases had a positive culture (20/52, 38.4%), 19.2% a positive ZN stain (10/52), and 26.2% a positive PCR (11/42). Sensitivity and specificity of PCR compared to cultures were 64% (95% CI: 35.14–87.24; 9/14) and 92% (95% CI: 73.9–99; 23/25), respectively. The sensitivity and specificity of ZN were 53% (95% CI 28.9–75.5; 10/19) and 100% (95% CI: 88.4–100; 30/30), respectively.

Microbiological investigations were additionally performed on BAL (8/554, 1.44%), stool (4/554, 0.72%), tissue biopsy (22/554, 3.9%), urine (68/554, 12.3%) and other biological fluids such as pleural, synovial and cerebrospinal fluid (19/554, 3.4%). No microbiological data were available for 73 children (13.2%). Considering children with TB meningitis, data about cerebrospinal fluid were known for 13 children. ZN staining was positive in 18.2% (2/11) of the cases, PCR in 57.1% of the cases (4/7), and cultures in half of the cases (6/12).

### 2.4. Sequelae

The outcome was known in 507/554 patients (91.5%), since 47 children were lost at follow-up. Thirty-six children with active TB (7.1%) developed sequelae. Half of the patients with CNS involvement (*n* = 11, 50%) exhibited persistent neurological manifestations (epilepsy, neurocognitive impairment, blindness, deafness, hemiplegia or hemiparesis). In 81.9% of osteoarticular TB, sequelae such as deformity, scoliosis and lameness were reported.

### 2.5. Treatment

#### 2.5.1. Active TB

The treatment of active TB cases was known for 534 (96.4%) patients. The details on the drug used, including the median dosage, median length of treatment, and side effects are reported in Table 5.

Overall, 210 (210/534; 39.3%) children were treated with three first-line drugs (isoniazid, rifampicin, pyrazinamide or ethambutol), whereas 216 (216/534; 40.4%) with four first-line drugs (isoniazid, rifampicin, pyrazinamide and ethambutol).

Fluoroquinolones were used in 42 children (42/534; 7.9%) and in particular the most used was moxifloxacin (31/42, 73.8%), which was given for a median length of 161 days. Injectable antitubercular drugs were administered in 48 children (48/534; 9%): streptomycin in 35 patients, amikacin in 12 patients, and kanamycin in one case. At least one oral bacteriostatic second-line antitubercular medication was used in 23 patients, cycloserine in half of the cases. Moreover, two antitubercular drugs, linezolid and clarithromycin, defined by WHO with unclear efficacy in MDR-TB treatment were used in 22 cases. Second-line drugs were frequently used in children without known resistances to anti-tubercular treatment (moxifloxacin: 19/31, 61.3%; linezolid 9/17, 52.9%; amikacin: 6/12, 50%; and cycloserine: 3/11, 27.3%). Side effects were reported in 35 patients. The most common one was the liver function impairment (hepatitis or hypertransaminasemia), reported in 54.3% of the cases (19/35).

Steroids were administered in 87 (16.3%) children, 38% of those received prednisone and 40% methylprednisolone. Overall, steroids in addition to the anti-tubercular treatment were used in 14 children with central nervous system (CNS) involvement, one pleuritis, one pericarditis and three miliary TB. Steroids were also used in 63 (72.4%) patients with pulmonary TB.

Regarding TB of the CNS, the treatment choice was known for 91% cases. In nine children, four first-line antitubercular drugs were used. An aminoglycoside was added to the standard treatment in five patients, in two patients a fluoroquinolone was used and cycloserine in two others.

#### 2.5.2. Drug Resistant TB

**Figure 1:**
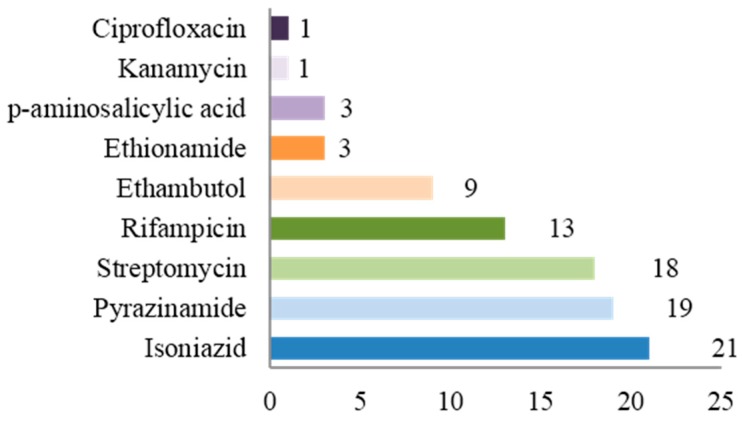
Resistances per single drug (*n*).

Thirty-nine (7%) children were infected with drug-resistant strains of *Mycobacterium tuberculosis*. Ten of them (25.9%) had MDR-TB and one (2.6%) had XDR-TB. In eight patients, the source case was known to have drug resistant TB. Thirty (76.9%) cases had pulmonary TB and the median age was 38 months. In our population, 15.4% of children with drug-resistant TB were Italian, whereas the country of origin was Eastern Europe in eight children, South America in eight, Africa in seven and Asia in 10 cases. Resistances per single drug are shown in Figure 1.

Six MDR-TB cases (60%) were treated with at least four second-line medications including an injective drug.

#### 2.5.3. Latent TB

Overall, the treatment of latent TB was known for 484 children (81.5%). Isoniazid alone was used in half of the cases (*n* = 264, 54.5%), with a median length of treatment of 190 days and a median daily dose of 10.1 mg/kg. In 220 children (45.5%), a combination of isoniazid and rifampicin was used with a median length treatment of 97 days. Side effects were lower in children treated with an isoniazid and rifampicin combination compared to isoniazid monotherapy (1/220, 0.45% *vs.* 5/264, 1.9%); however, this result was not statistically significant (*p* = 0.3).

## 3. Discussion

A picture of the burden of TB and pediatricians’ approach in a European country with low-TB incidence is reported in this retrospective study from the Italian Register of Pediatric Tuberculosis. Our Italian Register collects data on active and latent TB cases, TB contact and adopted/immigrated children screened for TB. Previous regional data were available from different local institutions [4,5,6,7]. Data from 31 Tuscan hospitals (by International Classification of Diseases-9 (ICD-9) code from the whole Tuscany region) for the years 1997–2011 were reviewed showing an increased TB incidence in children <5 years of age reaching 13.3 (95% CI: 7.8–18.9; *p* < 0.0001) per 100,000 in 2011 compared to 2007 [6]. A study limited to two pediatric centers in Rome retrospectively reviewed data of 214 children with definite or probable active TB from 1990 to 2009 [4]. In this study, almost 70% of the cases occurred in children below five years of age and more than half of the cases were among immigrants [4]. The epidemiology of TB in Emilia Romagna has been evaluated though a Regional TB surveillance system, including adult and childhood cases from 1996 to 2006 [7]. This study underlines immigration as an important risk factor for TB in low-incidence TB areas, with a reduction of TB cases born in Italy and an increase of cases in those born abroad [7]. In our study, within the active TB group, two-thirds of the cases occurred in children from a foreign country, considering immigrants as those who had at least one immigrant parent. These data overlap with the regional Italian data and national surveillance report. The definition of immigrants in TB reports is still debated, differently attributing the case origin based on the country of birth or family ethnicity [8,9]. In our study, the group resulting to have a higher TB incidence was the group of children born in Italy from foreign parents (44.4%). Similar findings were also found in an American study where the TB rate was estimated on the basis of the birth origin of children and parents, reporting that young children who were USA-born of foreign-born parents had relatively high rates of TB (53.3%) [8,9]. These findings could be mainly interpreted as the result of unvaccinated children exposed to adults with a high TB-risk, often living in poor conditions, or travelling to high incidence countries. Therefore, recent literature suggests that high-risk children should be vaccinated with BCG, as is already done in some European countries (*i.e.*, United Kingdom, France, and Scandinavian countries) [10,11,12,13,14]. In our study, the protective role of the BCG vaccination was not performed by a multivariate analysis because there was a high proportion of missing data on the BCG vaccination. Similarly, the finding of a higher percentage of BCG vaccinated children in the extra-pulmonary TB group (16.5% *vs.* 9.8%, *p* = 0.118), which is in contrast with the current evidence of protection of TB meningitis in children vaccinated with BCG, is probably due to the lack of data regarding BCG vaccinations in a high proportion of children. We also found that about one-third of active TB occurred in Italian children. This is an important finding for pediatricians, since in these cases TB is often diagnosed with a delay because of the lack of epidemiological suspicion.

Of note, approximately one-third of active TB was asymptomatic at presentation as it often occurs as the result of screening investigations among TB contacts.

The main problem in diagnosing childhood TB is related to its paucibacillary nature [8]. In our study the sensitivity and specificity of microbiological tests were sub-optimal, in line with literature findings [15,16,17,18]. TST and QFT-G-IT showed quite good sensitivity and specificity and a very low rate of indeterminate results similarly to what was already found in a multicenter study in Italy on a selected young subgroup of children from most Italian centers also present in the current Register [19]. Therefore, even if our multicenter study was not specifically designed to investigate the performance of IGRAs, we can conclude that the combination of these tests become of a very large use in our setting. In fact, guidelines recommend that combinations of the available tests (immunological, radiological and microbiological) be performed in children in order to increase the diagnostic rate [20,21,22,23].

The institution and development of a national Register responds to the need of monitoring childhood TB, considering that it is a potential pool for disease in adulthood and represents itself as a sentinel of disease in adult source cases. The inclusion of latent TB cases in our Register is pivotal considering that latent TB notification is not mandatory and that there is a high risk of progression in children, which is estimated to be up to 40%–50% below two years of age [23]. Therefore, in our study the median age of children with active TB resulted significantly lower than latent TB (*p* < 0.0001), confirming that the rate of progression is inversely related to age [24]. The treatment of latent TB in half of the cases in our study (*n* = 220) was done with a combination of isoniazid and rifampicin for three months, with low side effects. Currently, this combination is considered safe, with a higher completion rate, and as effective as six or nine months of isoniazid monotherapy [25].

Data on drug-resistant childhood TB in Italy are limited to case series [26,27,28,29,30] and related to adult rates [3]. In our study, the multidrug resistant TB rate was 7%, in line with the European data [3]. MDR-TB was in 25.9% of cases, whereas XDR-TB was in one case. Considering their risk factors for drug resistant TB (8/39, 20.5%), a contact with a MDR-TB source case was known in eight children and in about 85% of the cases the country of origin was with a high prevalence of resistant TB strains. It is important to remember that in children it is often difficult to grow mycobacterium, therefore, the data regarding drug sensitivities underestimate the real entity of the problem. Thus, it is mandatory to obtain information on the drug sensitivities of the index case [31].

Second-line drugs were used in 87 children with active TB (87/534; 16.3%). This proportion is higher than expected considering that drug resistant strains were only identified in 39 children. For example, at least one injectable anti-TB drug was used in five patients for the treatment of CNS TB, whereas a fluoroquinolone was used in two cases and cycloserine in two other cases. It can be hypothesized that second-line drugs were added to standard treatment in children with a complicated disease. However, the type of data available does not consent to clearly establish this correlation in our study. Despite the lack of standardized protocols for the therapy of drug-resistant TB in childhood, the treatment regimens are often empiric and based on a drug susceptibility pattern and disease severity, and the drugs used for children are often off-label and lack pediatric formulation [32,33].

Our study has several limits. Firstly, data do not result from a surveillance system, differently from other European studies carried out in Switzerland, England, and Poland, which are, however, limited to active TB, which is mandatory reported [34,35,36].

A second limitation is the high proportion of missing data, including follow-up data. However, this limitation is unfortunately common in all large retrospective multicenter studies. The retrospective monitoring is itself a limitation due to the lack of incidence data.

Improving the surveillance of childhood TB is important for healthcare workers and pediatricians. On a public health level, the understanding of the burden of the disease in the pediatric population can guide policy decisions and define priorities. Moreover, better knowledge of the distinctive aspects of childhood TB can improve the daily care of affected children. Since in our study more than 45% of active TB cases occurred before four years of age with a significantly higher proportion of CNS TB in this age group, future efforts should concentrate on the implementation of diagnostic tools and active surveillance, perhaps also extending the notification of latent TB in younger children.

## 4. Methods

A multicenter study was carried out in 27 pediatric hospitals, pediatric wards, and public health centers in Italy (see Supplementary Material Form). Data were collected retrospectively covering the period of time between 1 January 2010 and 31 December 2012 through a standardized form (Supplementary Material Form) and shared on the Italian Society of Pediatric Infectious Diseases network. The study was approved by the Institutional Review Board at the Department of Health Sciences, University of Florence (N. 32/14, approved on 12 November 2014). The aim of this study was to obtain epidemiologic and clinical data on childhood TB by creating and implementing the Italian Register of Pediatric Tuberculosis.

### 4.1. Study Design and Definitions

We retrospectively reviewed records of children (aged <18 years) diagnosed with active and latent TB between January 2010 and December 2012. Data on recently immigrated children screened for TB following a suspect or confirmed TB contact were also included in the study. For the purpose of this study, we considered immigrant children as those born abroad or in Italy from immigrant parents.

The following data were collected: demographic characteristics (*i.e.*, name and surname initials), clinical history including the period of observation, microbiologic results, imaging, medications and outcome. These data were retrospectively and anonymously collected for clinical purposes, therefore ethical committee approval was not requested.

All results were recorded in the study database following the international standards for the protection of privacy and personal information.

A positive tuberculin skin test (TST) was defined as an induration of 5 mm or greater in children with a suspected or confirmed TB contact or in children suspected to have TB or who are receiving immunosuppressive therapy. An induration of 10 mm or greater was considered positive in children younger than age 4 or in children with likely exposure to the TB disease. TST was defined positive with an induration of 15 mm or greater in children age 4 or older without any risk factors [24].

The interferon-gamma release assay (IGRA) performed was the QuantiFERON-TB Gold-in-tube (QFT-G-IT, Cellestis, Victoria, Australia). QFT-G-IT was considered positive when the concentration of the IFN-γ after stimulation with *Mycobacterium tuberculosis* antigens was ≥0.35 IU/mL. Only a small percentage of children in few centers were also tested by means of T-SPOT.TB (T-SPOT; Immunotec, Oxford, UK). Therefore, the results by this assay are not reported here. This study was not specifically designed for the purpose of evaluating the performance of QFT-G-IT and TST on TB. However, we calculated the sensitivity and specificity of TST QFT-G-IT on active TB. For this purpose, TST and IGRA were not included in the case definition.

Due to the paucibacillar nature of active TB in children, sensitivity for the diagnosis of active TB was calculated on microbiological confirmed TB, but also on probable TB, as previously in pediatric studies [37]. In particular, probable TB was defined on the basis of at least three among these findings: (1) exposure to an active TB case; (2) signs/symptoms suggestive of TB; (3) radiological findings consistent with pulmonary or extra-pulmonary TB; and (4) response to an adequate anti-tubercular treatment. Specificity of QFT-G-IT in the diagnosis of active TB was calculated defining children without active TB as those who had been investigated because of signs/symptoms suspect of TB and had some risk factors for active TB, which was reasonably excluded.

Active TB was defined as the presence of at least one clinical specimen (sputum, gastric aspirate/lavage or other biologic samples) positive for *Mycobacterium tuberculosis* on culture, microscopy, or nucleic acid amplification. An active TB diagnosis was also assigned to children with radiological and clinical findings consistent with active TB and with either exposure to a known TB case or with a positive TST and/or IGRA [24]. According to the country of origin, active TB cases were divided into three groups: born in Italy from Italian parents, born in Italy from foreign parents, and born abroad from foreign parents. The presence of a positive TST or an IGRA with normal physical and radiological findings was consistent with the definition of latent TB. Patients with negative TST or IGRA and no clinical and radiological signs of TB were considered uninfected. MDR-TB was defined as a TB infection or disease caused by a strain resistant to at least isoniazid and rifampicin. XDR-TB was defined as a TB infection or disease caused by a strain resistant to isoniazid, rifampicin, at least one fluoroquinolone, and one intravenous drug (amikacin, kanamycin, or capreomicin) [24].

### 4.2. Statistical Analysis

Statistical analysis was performed using SPSS (version 22.0, SPSS Inc., Chicago, IL, USA). Metric data were tested for normal distribution. Results were expressed as mean (standard deviations (SD)) or median (interquartile ranges (IQR)) as appropriate. Unpaired *t* test and Mann–Whitney tests were used to compare variables between groups. The χ-square test or Fisher test was performed when appropriate.

## Figures and Tables

**Table 1:** Main characteristics of children enrolled.

Patients Characteristics	Uninfected *n* = 3086	Latent TB ^1^ *n* = 594	Active TB *n* = 554	All *n* = 4234
Sex				
Male	1687/308 (54.7%)	329/592 (55.6%)	256/552 (46.4%)	2272/4227 (53.7%)
Female	1396/3083 (45.3%)	263/592 (44.4%)	296/552 (53.6%)	1955/4227 (46.3%)
Age (months)—Median (IQRs) ^2^	68 (34–114)	110 (65–173)	59 (20–129)	72 (35–124)
Age distribution				
≤1 year	248/3072 (8.1%)	13/594 (2.2%)	83/554 (15%)	344/4219 (8.2%)
1–4 years	841/3072 (27.4%)	90/594 (15.2%)	167/554 (30.2%)	1098/4219 (26%)
4–13 years	574/3072 (51.2%)	301/594 (50.7%)	216/554 (39.1%)	2091/4219 (49.6%)
≥13 years	409/3072 (13.3%)	190/594 (32%)	87/554 (15.7%)	686/4219 (16.3%)
Status				
Dead	0	0	2/554 (0.4%)	2/4234 (0.1%)
Lost to follow-up	42/3086 (1.4%)	45/594 (7.6%)	47/554 (8.5%)	134/4234 (3.2%)
Transferred to another center	0	0	7/554 (1.3%)	7/4234 (0.2%)
TB resolution	0	549/594 (92.4%)	498/554 (89.9%)	4091/4234 (96.6%)
Reason for investigation				
Adoption/immigrant screening	1949/3086 (63.2%)	359/594 (60.4%)	31/553 (5.6%)	2339/4233 (55.3%)
Contact with suspected/confirmed source case	976/3086 (31.6%)	222/594 (37.4%)	294/553 (53.2%)	1492/4233 (35.2%)
Symptomatic	161/3086 (5.2%)	13/594 (2.2%)	227/553 (41%)	401/4233 (9.5%)
Screening for use of biologic drugs	0	0	1/553 (0.2%)	1/4233 (0.1%)
Country of origin				
Asia	751/3086 (24.4%)	119/594 (20%)	77/554 (13.7%)	945/4234 (22.4%)
South-central America	493/3086 (16%)	71/594 (12%)	53/554 (9.6%)	617/4234 (14.6%)
East Europe	590/3086 (19.1%)	175/594 (29.5%)	138/554 (24.9%)	903/4234 (21.3%)
North America	1/3086 (0.1%)	0/594	0	1/4234 (0.1%)
North Africa	217/3086 (7%)	64/594 (10.8%)	74/554 (13.4%)	355/4234 (8.4%)
Sub-saharian Africa	322/3086 (10.4%)	88/594 (14.8%)	51/554 (9.2%)	461/4234 (10.9%)
Italy	692/3086 (22.4%)	70/594 (11.8%)	152/554 (27.4%)	914/4234 (21.6%)
Unknown	20/3086 (0.6%)	7/594 (1.2%)	9/554 (1.6%)	36/4234 (0.9%)
Source case				
Unknown	2584/3086 (83.7%)	480/594 (80.8%)	321/554 (57.9%)	3385/4234 (79.9%)
Household	150/3086 (4.8%)	70/594 (11.7%)	138/554 (24.9%)	358/4234 (8.4%)
Family member not household	111/3086 (3.5%)	27/594 (4.5%)	53/554 (9.5%)	191/4234 (4.5%)
Other not household	241/3086 (7.8%)	17/594 (2.8%)	29/554 (5.2%)	300/4234 (7%)
BCG ^3^ vaccination status				
Unknown	698/3046 (22.9%)	165/594 (27.8%)	223/549 (40.6%)	1086/4189 (25.9%)
Negative	1086/3046 (35.7%)	137/594 (23.1%)	264/549 (48.1%)	1487/4189 (35.5%)
Positive	1262/3046 (41.4%)	292/594 (49.2%)	62/549 (11.3%)	1616/4189 (38.6%)
Scar				
Unknown	682/2626 (26%)	179/523 (34.2%)	240/525 (45.7%)	1101/3674 (30%)
Negative	1018/2626 (35.7%)	160/523 (30.6%)	240/525 (45.7%)	1418/3674 (38.6%)
Positive	926/2626 (35.3%)	184/523 (35.2%)	45/525 (8.6%)	1155/3674 (31.4%)

^1^ TB: Tuberculosis; ^2^ IQR: Interquartile Range; ^3^ BCG: Bacillus Calmette–Guerin.

**Table 2:** Clinical signs/symptoms of the active tuberculosis cases.

Clinical Signs/Symptoms *	*n*
Fever	207
Cough	173
Lymphnode involvement	49
Weight loss	43
Gastrointestinal symptoms (nausea, vomiting, abdominal pain)	35
Respiratory symptoms (chest pain, dyspnea)	31
Osteoarticular symptoms (lameness, arthralgia)	31
Central nervous system involvement	17
Sweating	10
General malaise	9
Erythema nodosum	8
Hemoptysis	7
Conjunctival hyperemia	2
Anemia	1
Amenorrhea	1
Epistaxis	1
Microhematuria	1

* More than one sign/symptom is reported.

**Table 3:** Distribution of TB localization by age groups.

TB ^1^ Localization	≤4 Years	%	>4 Years	%	*p*	AOR ^2^ (95% CI) ^3^
Pulmonary TB	205/432	47.5	227/432	52.5	0.56	0.670 (0.444–1.012)
Lymph node TB	13/51	25.5	38/51	74.5	0.003	2.625 (1.366–5.047)
CNS ^4^ TB	16/22	72.7	6/22	27.3	0.009	0.297 9 (0.114–0.770)
Bone TB	11/24	45.8	13/24	54.2	0.958	0.978 (0.430–2.223)
Other site TB	7/35	20	28/35	80	0.002	3.549 (1.523–8.270)
Genitourinary tract TB			1/1		n.a. ^5^	n.a.

^1^ TB: tuberculosis; ^2^ AOR: adjusted odd ratio; ^3^ CI: confidence interval; ^4^ CNS: central nervous system; ^5^ n.a.: not applicable.

**Table 4:** Tuberculin skin test and QuantiFERON-TB Gold-in-tube (QFT-IT) results according to tuberculosis (TB) status.

Diagnostic Tests	Active TB (*n* = 554)	Latent TB (*n* = 594)
Tuberculin skin test		
<5 mm	82 (14.8%)	65 (10.9%)
≥5 mm	472 (85.2%)	529 (89.1%)
QuantiFERON-TB Gold-in-tube *		
Negative	36 (8.3%)	190 (34.3%)
Positive	388 (89.8%)	362 (65.4%)
Indeterminate	8 (1.8%)	1 (0.2%)

* QFT-IT results were not available in 122/554 (22%) children with active TB and 41/594 (6.9%) with latent TB. Results are reported in the table on 432 children with active TB and 553 with latent TB for whom QFT-IT results were known.

**Table 5:** Active TB treatment.

Drug	*n*	Median Dosage (mg/kg/day)	Median Length of Treatment (Days)	Side Effects (*n*)
Rifampicin	415	17.5	240	Itch, vomit and headache (1); rash (1); hypertransaminasemia (4)
Pyrazinamide	411	27	95	Hypertransaminasemia (7); vomit and malaise (1); epigastric pain (1); hyperuricemia (5); drug induced hepatitis (1)
Isoniazid	396	10	242	Drug induced hepatitis (1); hypertransaminasemia (4)
Ethambutol	245	17	86	Vomit and malaise (1); neutropenia (1); drug induced hepatitis (1); epigastric pain (1)
Streptomycin	35	19	31	
Moxifloxacin	31	10.3	161	Tendinopathy (1); long QT syndrome (1)
Linezolid	17	19.1	108	
Amikacin	12	16.8	24,5	
Cycloserine	11	14.1	220	
Levofloxacin	7	11.6	202	
PAS ^1^	6	118	172.5	
Ethionamide	6	15.4	91.6	
Clarithromycin	5	15.6	39.6	
Kanamicina	1	10	142	
Ciprofloxacin	4	17	238	Vomit and weight loss (1)

^1^ PAS: *p*-aminosalicylic acid.

## Supplementary Materials

Supplementary materials can be found at http://www.mdpi.com/1422-0067/17/6/960/s1.

## References

[1] World Health Organization. http://apps.who.int/iris/bitstream/10665/137094/1/9789241564809_eng.pdf?ua=1.

[2] Seddon J.A., Shingadia D. (2014). Epidemiology and disease burden of tuberculosis in children: A global perspective. Infect. Drug Resist..

[3] European Centre for Disease Control and Prevention Tuberculosis Surveillance and Monitoring in Europe, 2015. http://ecdc.europa.eu/en/publications/Publications/tuberculosis-surveillance-monitoring-Europe-2015.pdf.

[4] Buonsenso D., Lancella L., Delogu G., Krzysztofiak A., Testa A., Ranno O., D’Alfonso P., Valentini P. (2012). A twenty-year retrospective study of pediatric tuberculosis in two tertiary hospitals in Rome. Pediatr. Infect. Dis. J..

[5] Mammina C., Bonura C., Barchitta M., Quattrocchi A., Palermo M., Agodi A. (2014). Tuberculosis surveillance in Sicily, Italy. Epidemiol. Prev..

[6] Chiappini E., Bonsignori F., Orlandini E., Sollai S., Venturini E., Galli L., de Martino M. (2013). Increasing incidence of tuberculosis in Tuscan youth, 1997 to 2011. Pediatr. Infect. Dis. J..

[7] Odone A., Riccò M., Morandi M., Borrini B.M., Pasquarella C., Signorelli C. (2011). Epidemiology of tuberculosis in a low-incidence Italian region with high immigration rates: Differences between not Italy-born and Italy-born TB cases. BMC Public Health.

[8] Winston C.A., Menzies H.J. (2012). Pediatric and adolescent tuberculosis in the United States, 2008–2010. Pediatrics.

[9] Pang J., Teeter L.D., Katz D.J., Davidow A.L., Miranda W., Wall K., Ghosh S., Stein-Hart T., Restrepo B.I., Reves R. (2014). Tuberculosis Epidemiologic Studies Consortium. Epidemiology of tuberculosis in young children in the United States. Pediatrics.

[10] Montagnani C., Esposito S., Galli L., Chiappini E., Principi N., de Martino M., Italian Pediatric TB Study Group (2016). Recommendations for pediatric tuberculosis vaccination in Italy. Hum. Vaccines Immunother..

[11] Atchison C.J., Hassounah S. (2015). The UK immunisation schedule: Changes to vaccine policy and practice in 2013/14. JRSM Open.

[12] Barsky E. (2015). 2015 vaccination calendar. Soins.

[13] Haverkate M., D’Ancona F., Giambi C., Johansen K., Lopalco P.L., Cozza V., Appelgren E. (2012). Mandatory and recommended vaccination in the EU, Iceland and Norway: Results of the VENICE 2010 survey on the ways of implementing national vaccination programmes. Euro Surveill..

[14] Trogstad L., Ung G., Hagerup-Jenssen M., Cappelen I., Haugen I.L., Feiring B. (2012). The Norwegian immunisation register—SYSVAK. Euro Surveill..

[15] Chiappini E., Montagnani C., Venturini E., de Martino M., Galli L. (2015). Advantages of polymerase chain reaction assay performed on gastric aspirates for rapid diagnosis of pulmonary tuberculosis in children in a low prevalence country. Pediatr. Infect. Dis. J..

[16] Kordy F., Richardson S.E., Stephens D., Lam R., Jamieson F., Kitai I. (2015). Utility of gastric aspirates for diagnosing tuberculosis in children in a low prevalence area: Predictors of positive cultures and significance of non-tuberculous mycobacteria. Pediatr. Infect. Dis. J..

[17] López Ávalos G.G., Prado Montes de Oca E. (2012). Classic and new diagnostic approaches to childhood tuberculosis. J. Trop. Med..

[18] Tortoli E., Urbano P., Marcelli F., Simonetti T.M., Cirillo D.M. (2012). Is real-time PCR better than conventional PCR for *Mycobacterium tuberculosis* complex detection in clinical samples?. J. Clin. Microbiol..

[19] Garazzino S., Galli L., Chiappini E., Pinon M., Bergamini B.M., Cazzato S., dal Monte P., Dodi I., Lancella L., Esposito S. (2014). SITIP IGRA Study Group. Performance of interferon-γ release assay for the diagnosis of active or latent tuberculosis in children in the first 2 years of age: A multicenter study of the Italian Society of Pediatric Infectious Diseases. Pediatr. Infec. Dis. J..

[20] National Institute for Health and Care Excellence. https://www.nice.org.uk/guidance/cg117/chapter/guidance.

[21] Venturini E., Remaschi G., Berti E., Montagnani C., Galli L., de Martino M., Chiappini E. (2015). What steps do we need to take to improve diagnosis of tuberculosis in children?. Expert Rev. Anti Infect. Ther..

[22] Oberhelman R.A., Soto-Castellares G., Gilman R.H., Caviedes L., Castillo M.E., Kolevic L., del Pino T., Saito M., Salazar-Lindo E., Negron E. (2010). Diagnostic approaches for pediatric tuberculosis by use of different specimen types, culture methods, and PCR: A prospective case-control study. Lancet Infect. Dis..

[23] Piccini P., Chiappini E., Tortoli E., de Martino M., Galli L. (2014). Clinical peculiarities of tuberculosis. BMC Infect. Dis..

[24] Pickering L.K., Baker C.J., Kimberlin D.W., Long S.S. (2012). Red Book^®^: 2015 Report of the Committee on Infectious Diseases.

[25] Gwee A., Coghlan B., Curtis N. (2013). Question 1: What are the options for treating latent TB infection in children?. Arch. Dis. Child..

[26] Mignone F., Codecasa L.R., Scolfaro C., Raffaldi I., Lancella L., Ferrarese M., Garazzino S., Marabotto C., Esposito S., Gabiano C. (2014). The spread of drug-resistant tuberculosis in children: An Italian case series. Epidemiol. Infect..

[27] Pinon M., Scolfaro C., Bignamini E., Cordola G., Esposito I., Milano R., Mignone F., Bertaina C., Tovo P.A. (2010). Two pediatric cases of multidrug-resistant tuberculosis treated with linezolid and moxifloxacin. Pediatrics.

[28] Esposito S., Bosis S., Canazza L., Tenconi R., Torricelli M., Principi N. (2013). Peritoneal tuberculosis due to multidrug-resistant *Mycobacterium tuberculosis*. Pediatr. Int..

[29] Garazzino S., Scolfaro C., Raffaldi I., Barbui A.M., Luccoli L., Tovo P.A. (2014). Moxifloxacin for the treatment of pulmonary tuberculosis in children: A single center experience. Pediatr. Pulmonol..

[30] Mauro M.V., Cavalcanti P., Ledonne R., Giraldi C., Sperlì D. (2012). Description of primary multidrug-resistant tuberculous meningitis in an Italian child. Microb. Drug Resist..

[31] Schaaf H.S., Garcia-Prats A.J., Hesseling A.C., Seddon J.A. (2014). Managing multidrug-resistant tuberculosis in children: Review of recent developments. Curr. Opin. Infect. Dis..

[32] Taneja R., Garcia-Prats A.J., Furin J., Maheshwari H.K. (2015). Pediatric formulations of second-line anti-tuberculosis medications: Challenges and considerations. Int. J. Tuberc. Lung Dis..

[33] Seddon J.A., Hesseling A.C., Marais B.J., McIlleron H., Peloquin C.A., Donald P.R., Schaaf H.S. (2012). Pediatric use of second-line anti-tuberculosis agents: A review. Tuberculosis.

[34] Oesch Nemeth G., Nemeth J., Altpeter E., Ritz N. (2014). Epidemiology of childhood tuberculosis in Switzerland between 1996 and 2011. Eur. J. Pediatr..

[35] Korzeniewska-Koseła M. (2015). Tuberculosis in Poland in 2013. Prz. Epidemiol..

[36] Smith C., Abubakar I., Thomas H.L., Anderson L., Lipman M., Reacher M. (2014). Incidence and risk factors for drug intolerance and association with incomplete treatment for tuberculosis: Analysis of national case registers for England, Wales and Northern Ireland, 2001–2010. Thorax.

[37] Mandalakas A.M., Detien A.K., Hesseling A.C., Benedetti A., Menzies D. (2011). Interferon-gamma release assays and childhood tuberculosis: Systematic review and meta-analysis. Int. J. Tuberc. Lung Dis..

