# Supplementary material for: Pediatric Tuberculosis in Italian Children: Epidemiological and Clinical Data from the Italian Register of Pediatric Tuberculosis
Source: Int J Mol Sci. 2016 Jun 17;17(6):960. doi: 10.3390/ijms17060960 (PMC4926492; doi:10.3390/ijms17060960)
Supplement: Supplementary file 1 [file ijms-17-00960-s001.pdf]

Italian Register of Paediatric Tuberculosis

Coordinating centre: Florence ______________ Rome _______________

Enrollment Form

Number ______________

Hospital: _________________________ Paediatrician ____________________

Signature ____________________

1. PERSONAL DATA

Name (initial): _________ Surname (initial): _________Sex: [ ] M [ ] F Date of birth: ______ / ______ / ______

Place of birth (State): _____________________________Residence (Province): __________________________________

Country of origin (of the child if foreign origin, of the parents if born in Italy): ________________________________

If foreign, in Italy from: _______ / _______ / _______

The child is: [ ] adopted [ ] immigrant (even if born in Italy with foreign parents) [ ] Italian

Is a relative of other cases already enrolled? Yes [ ] No [ ]

If **yes**, specify: Name (initial): ________Surname (initial): ________ Date of birth: ____ /_____ /____

Form number: _____________________Relationship:______________________________

2. ANAMNESTIC DATA

Date of first observation: _____ / _____ / _____ Date of last visit: _____ / _____ / _____

Diagnosis: [ ] latent TB [ ] active TB [ ] uninfected Date of diagnosis: _____ / _____ / ____

In case of active TB: [ ] pulmonary TB [ ] extrapulmonary TB, specify: __________________________________

[ ] lymphnodes

[ ] central nervous system, specify _______________________

[ ] osteoarticular apparatus, specify ______________________

[ ] genitourinary system, specify _________________________

[ ] other, specify ______________________________________

Status: [ ] alive [ ] dead [ ] lost to follow-up

Sequelae: [ ] no [ ] yes , specify: ___________________

Date of readmission in community (if active TB) _______ / _____ /_____

If dead: Cause of death: ______________________________Date of death: _____ / _____ / _____

BCG vaccination: [ ] Yes [ ] No [ ] not known

SCAR: [ ] Yes [ ] No [ ] not known

HIV status: [ ] Yes [ ] No [ ] not known

Other diseases: [ ] Yes (specify: ____) [ ] NO

Immunosuppressive therapy: [ ] Yes (specify: ___________________________) [ ] No

Reason for investigation:

[ ] Adoption/immigrant screening [ ] Screening for use of biologic drugs

[ ] Contact with suspected/confirmed source case [ ] Symptomatic

If symptomatic (indicate all the signs and symptoms present):

Signs/symptoms: [ ] cough [ ] weight loss [ ] fever [ ] hemoptysis [ ] lymphadenopathy

[ ] night sweats [ ] other, specify: ________________________

If known contact:

Type of relation with the source case: [ ] parent [ ] relative [ ] not household [ ] household

[ ] other, specify: ____________________

Contagiousness of the source case: [ ] ZN staining POS [ ] ZN staining NEG, colture POS [ ] both NEG

Source case country of origin: ____

Source case treatment: [ ] Yes [ ] No

Source case susceptibility test (highlight resistences):

[ ] isoniazid [ ] ethambutol [ ] pyrazinamide [ ] rifampicin [ ] streptomycin [ ] other

3. DIAGNOSIS

Tuberculine Skin Test (TST)

Date: _____ / _____ / _____ Millimeters of induration: _______

Reversion: [ ] Yes [ ] NO [ ] not available

IGRAs

Quantiferon: Type: [ ] TB Gold [ ] In-Tube

Data: _____ / _____ / _____ [ ] Positive [ ] Negative [ ] Indeterminate

IFNγ level after stimulation with: Mitogen: ______ UI/mL TB antigens: ______ UI/mL

Elispot: Date: _____ / _____ / _____

[ ] Positive [ ] Negative [ ] Borderline [ ] Indeterminate

MICROBIOLOGICAL DATA

Sample: [ ] Gastric aspirate [ ] Sputum [ ] BAL

	Date ____/____/____	Date ____/____/____	Date ____/____/____
	I sample	II sample	III sample
Colture	[ ] P [ ] N	[ ] P [ ] N	[ ] P [ ]N
ZN staining	[ ] P [ ] N	[ ] P [ ] N	[ ] P [ ]N
PCR	[ ] P [ ] N	[ ] P [ ] N	[ ] P [ ]N

If ZN staining positive, was the test repeated before community readmission? [ ] Yes [ ] No
If Yes, date of negative ZN staining:____/____/____

Other Samples:

	ZN staining	Colture	PCR
Pleural fluid: ___ /___ /___	[ ] P [ ] N	[ ] P [ ] N	[ ] P [ ] N
CSF: ___ /___ /___	[ ] P [ ] N	[ ] P [ ] N	[ ] P [ ] N
Lymphnode: ___ /___ /___	[ ] P [ ] N	[ ] P [ ] N	[ ] P [ ] N
Urine: ___ /___ /___	[ ] P [ ] N	[ ] P [ ] N	[ ] P [ ] N
Other:___ /___ /___	[ ] P [ ] N	[ ] P [ ] N	[ ] P [ ] N

Specify:(_______)

Antimicrobial Susceptibility Test (highlight resistences): [ ] Yes, data ___/___/___ [ ] No

Specify resistences, if applicable:

[ ] isoniazid [ ] ethambutol [ ] pyrazinamide [ ] rifampicin [ ] streptomycine [ ] other ___________

Blood tests

**Data**	**WB** cell $\times 10^3/\mu L$	**RBC** cell $\times 10^3/\mu L$	**Hb** g/dL	**E** cell/µL	**CD4+** cell/µL (%)	**γ-GT** UI/L	**ALT** UI/L	**AST** UI/L	**PCR** mg/dl	**25OHD** ng/ml

Radiological tests:

Chest X-ray: date: ___ /___ /___ [ ] not performed

[ ] negative

[ ] consolidation [ ] ilar congestion

[ ] calcifications [ ] cavitation

[ ] interstitial disease [ ] other, specify_____________

Lung-CT date: ___ /___ /___ [ ] not performed

Contrast: [ ] Yes [ ] No [ ] negative

[ ] consolidation [ ] ilar congestion

[ ] calcification [ ] cavitation

[ ] miliary TB [ ] atelectasis

[ ] other, specify: ___________________

Other radiological tests: [ ] Yes, specify: __________________________ [ ] No

Date ____/____/_____ Result: ____

4. Treatment [ ] **Prophylaxis** [ ] **Therapy** **weight (Kg):** ____________

Isoniazid ongoing: [ ] Yes [ ] No

Dosage (mg/kg/day): ___ from___ /___ /___ to: ___ /___ /___

Therapy discontinued for: [ ] end of therapy [ ] patient decision [ ] hypertransaminasemia

[ ] drug resistant TB [ ] rash [ ] other (specify) ______________

Rifampicin ongoing: [ ] Yes [ ] No

Dosage (mg/kg/day): ___ from___ /___ /___ to: ___ /___ /___

Therapy discontinued for: [ ] end of therapy [ ] patient decision [ ] hypertransaminasemia

[ ] drug resistant TB [ ] rash [ ] other (specify) ______________

Pyrazinamide ongoing: [ ] Yes [ ] No

Dosage (mg/kg/day): ___ from ___ /___ /___ to: ___ /___ /___

Therapy discontinued for: [ ] end of therapy [ ] patient decision [ ] hypertransaminasemia

[ ] drug resistant TB [ ] rash [ ] other (specify) ______________

Ethambutol ongoing: [ ] Yes [ ] No

Dosage (mg/kg/day): ___ from ___ /___ /___ to: ___ /___ /___

Therapy discontinued for: [ ] end of therapy [ ] patient decision [ ] hypertransaminasemia

[ ] drug resistant TB [ ] rash [ ] other (specify) ______________

Combination of antituberculosis drugs [es. Rifinah (HR), Rifater (HRP)]

Combination:________________ dosage (mg/kg/day): ___ from___ /___ /___ to: ___ /___ /___

Therapy discontinued for: [ ] end of therapy [ ] patient decision [ ] hypertransaminasemia

[ ] drug resistant TB [ ] rash [ ] other (specify) ______________

Steroid therapy ongoing: [ ] Yes [ ] No dosage (mg/Kg/day):_______ from ___/___/___ to___/___/___

Therapy discontinued for: [ ] end of therapy [ ] patient decision [ ] hypertransaminasemia

[ ] drug resistant TB [ ] rash [ ] other (specify) ______________

Other drugs:

________________________________ongoing: [ ] Yes [ ] No

Dosage (mg/kg/day): ___ from___ /___ /___ to: ___ /___ /___

Therapy discontinued for: [ ] end of therapy [ ] patient decision [ ] hypertransaminasemia

[ ] drug resistant TB [ ] rash [ ] other (specify) ______________

________________________________ongoing: [ ] Yes [ ] No

Dosage (mg/kg/day): ___ from___ /___ /___ to: ___ /___ /___

Therapy discontinued for: [ ] end of therapy [ ] patient decision [ ] hypertransaminasemia

[ ] drug resistant TB [ ] rash [ ] other (specify) ______________

________________________________ongoing: [ ] Yes [ ] No

Dosage (mg/kg/day): ___ from___ /___ /___ to: ___ /___ /___

Therapy discontinued for: [ ] end of therapy [ ] patient decision [ ] hypertransaminasemia

[ ] drug resistant TB [ ] rash [ ] other (specify) ______________
